# Effects of ATF2/TSC1 on epilepsy by modulating the microphages polarization of microglia

**DOI:** 10.1038/s41598-025-04914-4

**Published:** 2025-07-02

**Authors:** Wenjiao Huang, Wenli Chen, Zhong Zhao, Lingchun Liu, Yuanyuan Zhao, Xinzhang Chen, Rong Li

**Affiliations:** 1 School of Medicine, Kunming University of Science and Technology, No. 727 Jingming South Road, Kunming, 650500 Yunnan China; 2 Department of Neurology, The First People’s Hospital of Yunnan Province, No. 157 Jinbi Road, Kunming, 650034 Yunnan China

**Keywords:** Epilepsy, Activating transcription factor 2, Microglia, Microphages polarization, Tuberous sclerosis complex 1, Mechanisms of disease, Neurological disorders

## Abstract

Epilepsy (EP) is a chronic nervous system disease characterized by recurrent attacks, and its causes are complicated. Inflammatory reaction mediated by microglia is an important factor in the progression of EP. Activating transcription factor 2 (ATF2) can be used as a transcription factor to regulate the microglia-mediated inflammatory response, but its role in EP is unclear. In this study, kainic acid (KA) was used to induce the EP cell and mouse model. Real-time polymerase chain reaction was used to detect ATF2, TNF-α, IL-6, TGF-β, and IL-10 mRNA expression. ATF2, INOS, ARG1, and TSC1 protein levels was examined by western blot. The fluorescence intensity of ATF2, IBA1, CD80, and CD206 was examined by immunofluorescence staining. The cell ratios of CD80, IL-1β, CD206, and CD63 were detected by flow cytometry. Dual-luciferase reporter and chromatin immunoprecipitation assays were conducted to verify the interaction between ATF2 and TSC1. Hematoxylin & eosin and Nissl staining were used to observe the structure of hippocampus and Nissl bodies. The results indicated that KA induced M1 polarization of HMC3 cells and increased the levels of TNF-α and IL-6 mRNA by activating KA receptors, and inhibiting KA receptors attenuated the M1 polarization of KA-induced HMC3 cells. ATF2 expression was increased in KA-induced HMC3 cells and hippocampal tissues of mouse, while TSC1 expression was repressed. ATF2 knockdown diminished the M1 polarization of KA-induced HMC3 cells, enhanced the M2 polarization, and relieved neuroinflammation in EP mouse. TSC1 overexpression inhibited M1 polarization in KA-induced HMC3 cells. Dual luciferase and chromatin immunoprecipitation results revealed that ATF2 bound to the promoter of TSC1 and negatively regulated the transcription of TSC1. In conclusion, inhibition of ATF2 and promotion of TSC1 transcription may be a new pathophysiological mechanism for the treatment of EP neuroinflammation.

**Supplementary Information:**

The online version contains supplementary material available at 10.1038/s41598-025-04914-4.

## Introduction

Epilepsy (EP) is a nervous system disease with persistent epileptic tendency and corresponding neurobiological, cognitive, social and psychological characteristics^1^. Previous studies have shown that the imbalance between excitation and inhibition is the main pathogenic factor, and the imbalance between excitation and inhibition will cause abnormal discharge of neurons and lead to seizures^2^. There are about 70 million EP patients in the world, and there are as many as 10 million patients in China, and about 400 thousand people are added every year^3^. EP occurs in various forms, including generalized convulsion, loss of consciousness, abnormal behavior and sensory, emotional and cognitive dysfunction. EP has the characteristics of long course and recurrent seizures, most patients can effectively control their illness after treatment, but about 30% patients still develop intractable EP after regular treatment^4^. Therefore, it is urgent to study the potential pathogenesis of EP and find potential therapeutic targets.

Microglia are the main immune cells in the central nervous system, and maintain the steady state of the central nervous system through immune monitoring^5^. It is found that microglia are activated in EP^6^ which can stimulate the polarization of microglia into M1 type and M2 type. M1 microglia secrete pro-inflammatory cytokines TNF-α and IL-1β to aggravate the inflammatory reaction. M2 microglia secrete anti-inflammatory cytokine IL-10 to reduce inflammatory reaction^7^. Studies have shown that neuroinflammation may trigger EP^8^. Therefore, induction M2 microglia is helpful to alleviate EP. However, the specific mechanism of regulating microglia polarization is still unclear.

As a member of transcription factor family, activating transcription factor 2 (ATF2) can regulate cell survival and differentiation by combining with other transcription factor heterodimers. ATF2 can be used as an oncogene^9^ or tumor suppressor gene^10^ and also participate in regulating osteogenesis^11^ and inflammatory reaction^12^ et al. Isoflurane-activated ATF2 pathway could induce oxidative stress and inflammation in BV2 cells^13^. Li et al.^14^ reported that ATF2 promoted the inflammatory reaction of microglia, and inhibition of ATF2 significantly reduced the expression of pro-inflammatory cytokines in LPS-induced microglia. However, it is still unclear whether ATF2 regulates microglia polarization in EP.

In this study, we induced EP cell model and a mouse model through kainic acid (KA), and found that ATF2 expression was increased in human microglia (HMC3) and hippocampus of mouse induced by KA. Knocking down ATF2 repressed the M1 polarization of microglia in vitro and in vivo. Mechanistic study indicated that ATF2 served as a transcription factor and inhibited the transcription of TSC1.

## Materials and methods

### Cell culture and treatment

Human microglial clone 3 (HMC3) cells were kindly provided by Pricella Life Science & Technology Co., Ltd. The cells grown in minimum essential medium supplemented with nonessential amino acid, 10% fetal bovine serum, and 1% penicillin/streptomycin. HMC3 cells were cultured in a cell incubator at 37 °C. The 100 µM KA (Macklin, China) were used to treat HMC3 cells for 2, 4, and 6 h to induce the EP cell model^15–17^.

Before treating the cells with KA, HMC3 cells were exposed to 10 µg/mL and 100 µg/mL of the KA inhibitor topiramate (TPM, Medchem Express, NJ, USA) in a 37 ℃ incubator for 18 h of pretreatment. Following this, 100 µM of KA was added, and the incubation was continued for 6 h^18,19^.

### Cell transfection

sh-NC, sh-ATF2, pcDNA-NC, pcDNA-ATF2, pcDNA-TSC1 plasmids were synthesized by Hanheng biology science and technology co., ltd (Shanghai, China). According to the manufacturer’s introduction, Lipofectamine 2000 was used to transfect these plasmids into HMC3 cells. Western blot was performed to detect the transfection efficiency after 48 h.

### Real-time polymerase chain reaction (RT-qPCR)

TRIzol reagent (Shanghai yuanye, China) was used to extract total RNA from cells. cDNA was synthesized according to the manufacturer’s instructions of reverse transcription kit (Thermo Fisher Scientific, Waltham, MA, USA). RT-qPCR reaction was carried out using SYBR^®^ Premix Ex Taq™ GC kit (Hunan OBM, China) with cDNA as template. With GAPDH as internal reference, the relative expression of ATF2 mRNA was analyzed by 2^−ΔΔCt^ method.

### Western blot

RIPA reagent (Shanghai Beyotime, China) was used to extract total protein from cells. The protein concentration was examined using bicinchoninic acid method (Aidikang, China). Protein was separated by sodium dodecyl sulfate-polyacrylamide gel electrophoresis and transferred to polyvinylidene fluoride membrane (Sigma-Aldrich, St. Louis, MO, USA). The membrane was sealed with 5% skimmed milk for 2 h, then incubated overnight with mouse anti-GLUK2 (Abcam, UK; 1: 500), rabbit anti- GLUK5 (Abcam, UK; 1 µg/mL), ATF2 (Abcam, UK; 1: 1000), ARG1 (Beijing Bioss, China; 1: 1000), INOS (Beijing Bioss, China; 1: 300), TSC1 (Beijing Bioss, China; 1: 1000) and β-actin (Beijing Bioss, China; 1: 2000) antibodies, and then incubated with the second antibody (Goat Anti-Rabbit IgG H&L, 1: 1000; Goat Anti-Mouse IgG H&L, 1: 2000; Abcam, UK) at room temperature for 1 h after rinsing. Enhanced chemiluminescence reagent (Shanghai Beyotime, China) was added to visualize the protein bands, and the gray value was analyzed by Image J software.

### Immunofluorescence (IF) staining

HMC3 cells were inoculated into a 6-well plate (5 × 10^5^ cell/well). After transfection and KA treatment, the cells were fixed using 4% paraformaldehyde. Cells were blocked with 3% bovine serum albumin for 30 min and incubated with IBA1 (Proteintech, China; 1: 500) and ATF2 (Proteintech, China; 1: 100) primary antibody at 4 ℃ overnight. The rinsed cells were incubated with fluorescent labeled secondary (Abcam, UK; 1: 500) antibody at room temperature for 1 h, and DAPI was added to stain the nucleus. The expression of IBA1 and ATF2 was observed by a fluorescence microscope.

### Flow cytometry

After transfection and KA treatment, HMC3 cells were collected into centrifuge tubes. After centrifugation, cells were incubated with antibodies against CD80 (Abcam, UK; 1: 5000), IL-1β (Abcam, UK; 1: 500), CD206 (Beijing Bioss, China; 1 µg/10^6^ cells), and CD63 (Abcam, UK; 1: 100) in the dark for 20 min. After washing, the percentage of CD80, IL-1β, CD206, and CD63 positive cells was detected by flow cytometry.

### Chromatin Immunoprecipitation (CHIP)

The interaction between ATF2 and TSC1 promoters was verified by CHIP experiment. HMC3 cells were cross-linked and fixed with methanol. The cells were lysed with nuclear lysis buffer and treated with ultrasound. The supernatant of cell lysates was diluted using protease inhibitor buffer and incubated overnight with ATF2 antibody and magnetic beads. The next day, the magnetic beads were washed and incubated with elution buffer overnight. The DNA purification kit (Beijing Baiaolaibo, China) was used to purify DNA, and the enrichment of TSC1 promoter (P1, P2, and P3) was detected by RT-PCR.

### Luciferase reporter assay

TSC1 promoter and mutant promoter sequences were constructed and packaged using pGL4.10 plasmid. The empty, wild-type pGL4.10-TSC1, mutant pGL4.10-TSC1 promoter plasmid, and ATF2 overexpression plasmid were transfected into HMC3 cells. After 48 h, the luciferase activity was detected by dual luciferase reporter gene detection system (Shanghai Yeasen, China).

pcDNA3.1-HA empty vector, pcDNA3.1-HA-ATF2 (0.1, 1, and 2 µg) and pGL4.10-TSC1 were transfected into HMC3 cells, and the luciferase activity of pGL4.10-TSC1 reporter gene was detected by dual luciferase reporter gene detection system (Shanghai Yeasen, China).

### Induction of EP mouse and intrahippocampal injections

The animal experiments were approved by the Experimental Animal Ethics Committee of Yunnan Labreal Biotechnology Co., Ltd (PZ20231012) and performed in accordance with the Animal Research: Reporting of In Vivo Experiments (ARRIVE) guidelines 2.0. All methods were carried out in accordance with relevant guidelines and regulations.

Twenty-four male C57BL/6 mice (eight-week, 20–22 g) were provided by Hunan Silaike Jingda Experimental Animal Co., Ltd. All mice were adaptively fed for one week, and randomly divided into 4 groups: sham, KA, KA + sh-NC, and KA + sh-ATF2 (*n* = 6). The 3 µL of sh-NC (KA + sh-NC group) and sh-ATF2 (KA + sh-ATF2 group) packaged with adeno-associated virus were injected into the right hippocampus of mouse, and EP induction was performed one week later. The mouse was anesthetized with 40 mg/kg pentobarbital sodium and fixed on a stereotactic instrument. 0.9% sodium chloride was used to dilute KA to 83 ng/mL. The 3 µL KA (0.25 µg/mouse) diluted solution was injected into the right hippocampus (anteroposterior − 2.2 mm, mediolateral − 2.2 mm, dorsoventral − 1.8 mm) of mouse. The drug was slowly injected into mouse, and the needle remained in place for 10 min after injection. Sham group mice were injected with the same amount of normal saline as KA. One hours after the seizure of mouse, 10 mg/kg of diazepam were injected to terminate. Twenty-eight days later, Racine scale scores were used to evaluate the seizure severity of mice. Excessive pentobarbital sodium (90 mg/kg) was injected intraperitoneally to euthanize mice. The hippocampus of mouse was collected for detection.

According to Racine scale, the seizure status of mouse was evaluated. Grade 0: no response; grade I: facial clonus; grade II: grade I pluses rhythmic nodding; grade III: grade II pluses myoclonus of forelimbs, but no upright position of hind limbs; grade IV: grade III pluses upright position of hind limbs; grade V: generalized tonic-clonic seizure, loss of position control. A Racine score of III or higher lasting for more than 30 min is determined as an epileptic seizure.

### Hematoxylin & Eosin (HE)

The hippocampus of mouse was fixed with 4% paraformaldehyde (Shanghai Nuonin, China), embedded in paraffin and sliced (5 μm). Slices were treated with xylene and ethanol, dyed with hematoxylin and eosin (Wuhan Servicebio, China), rinsed, and dehydrated with ethanol. The hippocampal structure was observed under a microscope.

### IF staining

The sections of mouse hippocampus were soaked with 0.5% TrixonX-100, then incubated with 0.2% TrixonX-100 and 5% BSA, and incubated overnight at 4 ℃ with primary antibody of IBA1 (Proteintech, China; 1: 500), CD80 (Beijing Bioss, China; 1: 500), and CD206 (Proteintech, China; 1: 500), and incubated with second antibody (Abcam, UK; 1: 500) at room temperature for 1 h after rinsing. The nucleus was stained with DAPI. After the slices sealing, the expressions of IBA1, CD80, and CD206 were observed by a fluorescent microscope.

### Nissl staining

First, the slices of mouse hippocampus were baked in the oven, dewaxed with xylene, and dehydrated with ethanol. Next, the slices were put into toluidine blue solution (Shanghai Jinpan, China) for differentiation. The slices were washed with distilled water and then treated with ethanol and xylene. The Nissl bodies were observed under a microscope.

### Statistical analysis

GraphPad Prism 8.0 was used to analyse the data and graph. The measurement data passed normality test is expressed as mean ± standard deviation. Independent sample *t* test was used to analyze the difference between two groups, and one-way analysis of variance was used to compare the difference among multiple groups. *P* < 0.05 is considered statistically significant.

## Results

### KA-induced M1 polarization of microglia HMC3

**Fig. 1:**
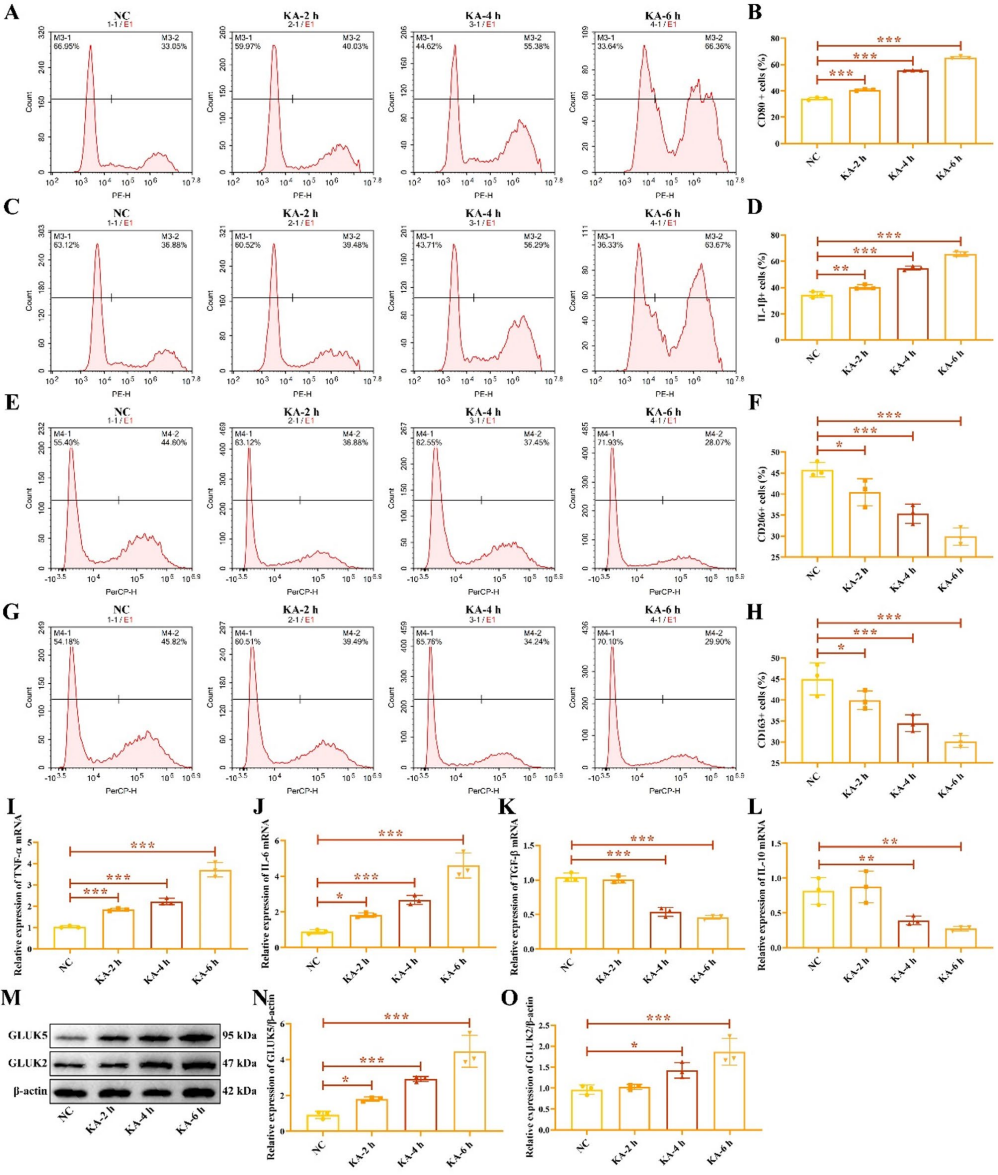
KA-induced M1 polarization of microglia HMC3. Flow cytometry was used to detect M1 macrophage markers CD80 (**A**-**B**) and IL-1β (**C**-**D**), as well as M2 macrophage markers CD206 (**E**-**F**) and CD163 (**G**-**H**). RT-qPCR was performed to measure the mRNA levels of inflammatory cytokines TNF-α (**I**), IL-6 (**J**), TGF-β (**K**), and IL-10 (**L**). Western blot analysis was conducted to assess the protein expression of KA receptors GLUK5 and GLUK2 (**M**-**O**). ^*^P < 0.05, ^**^P < 0.01, ^***^P < 0.001.

Currently, there is no unified protocol for inducing epileptic cell models with KA. Based on research reports and our previous research, we stimulated HMC3 cells with 100 µM KA for 2, 4, and 6 h to induce epileptic cell models. Flow cytometry analysis revealed that the positive rates of M1 macrophage markers CD80 (Fig. 1A-B) and IL-1β (Fig. 1C-D) increased with KA treatment duration, while the positive rates of M2 macrophage markers CD206 (Fig. 1E-F) and CD163 (Fig. 1G-H) decreased as KA treatment time increased. RT-qPCR demonstrated that with increasing duration of KA treatment, the mRNA levels of inflammatory cytokines TNF-α (Fig. 1I) and IL-6 (Fig. 1J) increased, while the levels of TGF-β (Fig. 1K) and IL-10 (Fig. 1L) decreased. Western blot analysis indicated that the expression of KA receptors GLUK5 and GLUK2 increased with prolonged KA stimulation of cells (Fig. 1M-O), the original gels were shown in the Supplementary Fig. 1. These data suggest that the M1 polarization state of HMC3 cells is more pronounced after 6 h of KA stimulation compared to 2 and 4 h, and we hypothesize that KA induces EP by activating KA receptors.

### Inhibition of KA receptor attenuates the M1 polarization effect of KA on HMC3 cells

**Fig. 2:**
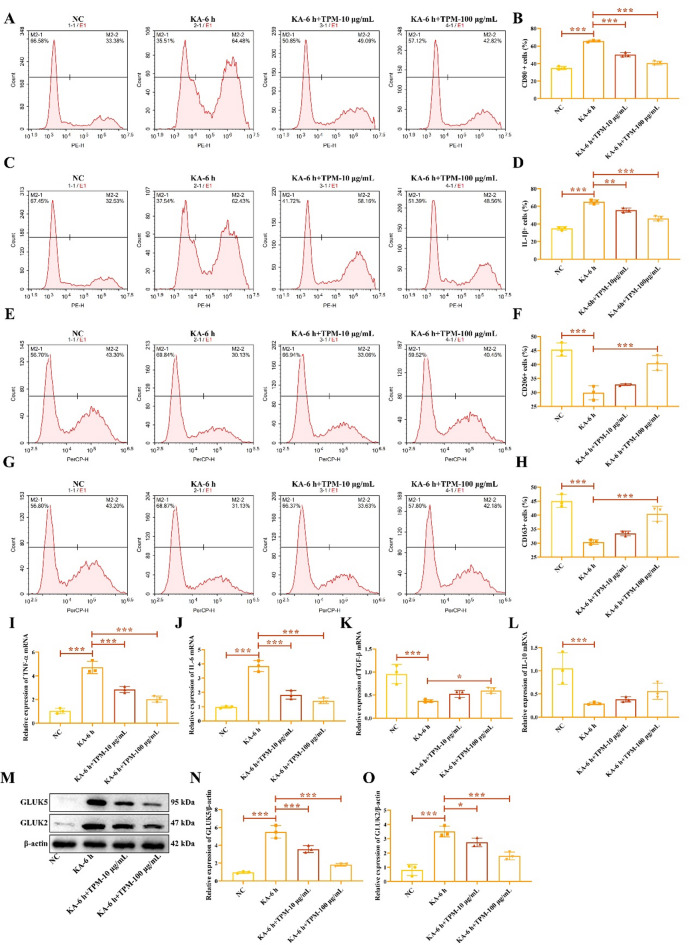
Inhibition of KA receptor attenuates the M1 polarization effect of KA on HMC3 cells. Flow cytometry was used to detect M1 macrophage markers CD80 (**A**-**B**) and IL-1β (**C**-**D**), as well as M2 macrophage markers CD206 (**E**-**F**) and CD163 (**G**-**H**). RT-qPCR was employed to assess the mRNA levels of inflammatory cytokines TNF-α (**I**), IL-6 (**J**), TGF-β (**K**), and IL-10 (**L**). Western blot analysis was performed to determine the protein expression of KA receptors GLUK5 and GLUK2 (**M**-**O**). ^*^P < 0.05, ^**^P < 0.01, ^***^P < 0.001.

Next, we treated KA-induced HMC3 cells with KA receptor inhibitors TPM to investigate the effect of KA receptor inhibition on KA-activated cells. Compared to the KA-6 h group, HMC3 cells treated with TPM showed a decrease in CD80 (Fig. 2A-B) and IL-1β (Fig. 2C-D) positive cells, and an increase in CD206 (Fig. 2E-F) and CD163 (Fig. 2G-H) positive cells. Additionally, TPM treatment reduced the mRNA levels of pro-inflammatory cytokines TNF-α (Fig. 2I) and IL-6 (Fig. 2J), while increasing the mRNA levels of anti-inflammatory cytokines TGF-β (Fig. 2K) and IL-10 (Fig. 2L). Western blot analysis revealed that the expression of GLUK5 and GLUK2 was significantly lower in the TPM group compared to the KA-6 h group (Fig. 2M-O), and the inhibitory effect was more pronounced with 100 µg/mL TPM. The original gels were shown in the Supplementary Fig. 2. These findings reveal KA induces M1 macrophage polarization in HMC3 cells through the activation of KA receptors.

### ATF2 is overexpressed in EP cell model

**Fig. 3:**
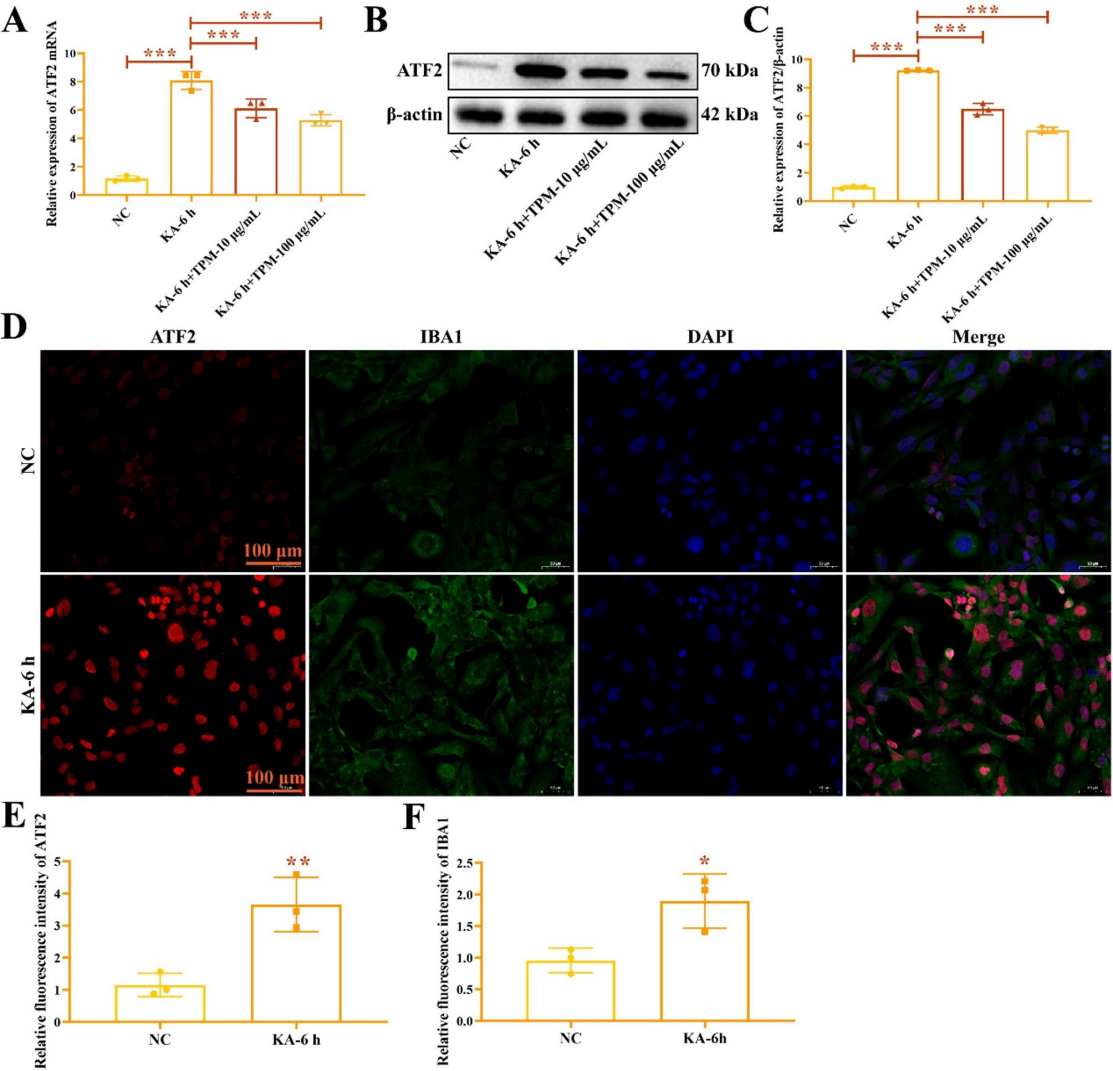
ATF2 is overexpressed in KA-induced HHMC3 cells. (**A**) ATF2 mRNA expression in KA-induced HMC3 cell. (**B**-**C**) ATF2 protein expression in KA-induced HMC3 cell. (**D**). The fluorescence intensity of ATF2 and IBA1. Analysis of the relative fluorescence intensity of ATF2 (**E**) and IBA1 (**F**). ^*^P < 0.05, ^**^P < 0.01, ^***^P < 0.001.

Research suggests that microglial activation is involved in the occurrence and development of EP^20^and the expression level of ATF2 is closely related to the behavior of microglia^21^. However, the role of ATF2 in regulating microglia during EP has not been reported in previous studies. In this part of the study, we utilized a KA-induced EP cell model to investigate the expression of ATF2 in the EP. The mRNA expression of ATF2 in KA-induced HMC3 cells was increased than in NC group (Fig. 3A). Western blot results indicated that ATF2 protein was upregulated by KA (Fig. 3B-C), the original gels were shown in the Supplementary Fig. 3. Treatment with TPM inhibited the expression of ATF2 mRNA and protein (Fig. 3A-C). IF staining showed that the induction of KA increased the fluorescence intensity of ATF2 (Fig. 3D-E) and IBA1 (Fig. 3D and F). These results demonstrate that ATF2 is increased in KA-induced EP cell model.

### ATF2 inhibition represses the M1 polarization of HMC3 cells

**Fig. 4:**
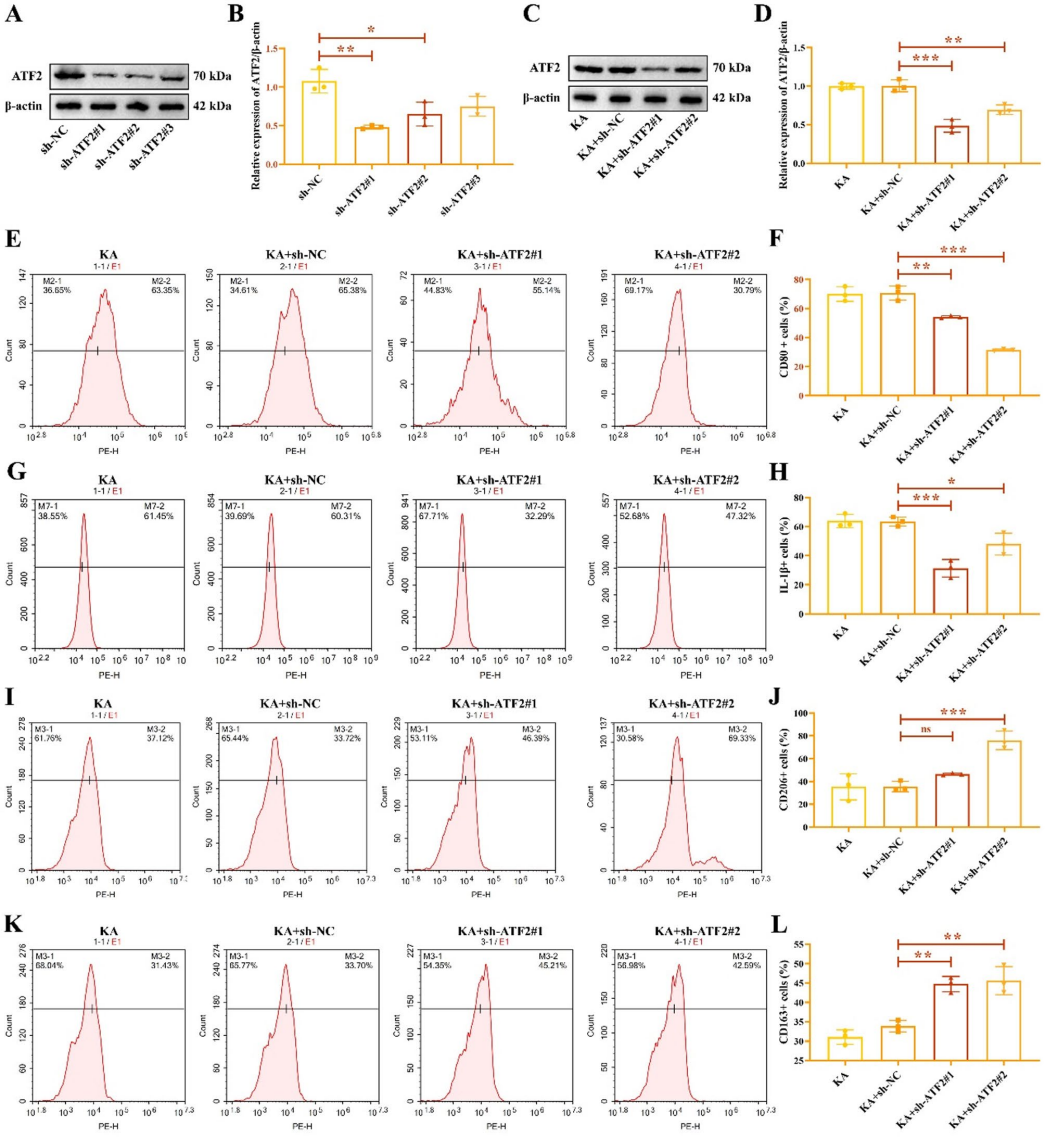
ATF2 inhibition represses the M1 polarization of HMC3 cells. (**A**-**B**) ATF2 protein level in HMC3 cells transfected with sh-ATF2. (**C**-**D**) ATF2 protein expression in KA-induced HMC3 cells transfected with sh-ATF2. Flow cytometry was performed to access cell ratio of M1 microphage CD80 (**E**-**F**), IL-1β (**G**-**H**) and M2 microphage marker CD206 (**I**-**J**), CD163 (**K**-**L**). ^*^P < 0.05, ^**^P < 0.01, ^***^P < 0.001.

Next, we transfected sh-ATF2 into HMC3 cells to explore the function of ATF2 in microglia. The results were showed in Fig. 4A-B, ATF2 expression was significantly decreased in HMC3 cell transfected with sh-ATF2#1 and sh-ATF2#2. The original gels were shown in the Supplementary Fig. 4. In KA-induced HMC3 cells, transfection with sh-ATF2 decreased ATF2 protein level (Fig. 4C-D). Flow cytometry assay was carried out to examine the ratio of M1/M2-type microphages, and results found that the positive proportion of M1 macrophage markers CD80 (Fig. 4E-F) and IL-1β (Fig. 4G-H) were decreased in ATF2 inhibition group. However, the positive proportion of M2 macrophage markers CD80 (Fig. 4I-J) and IL-1β (Fig. 4K-L) were enhanced in ATF2 inhibition group.

**Fig. 5:**
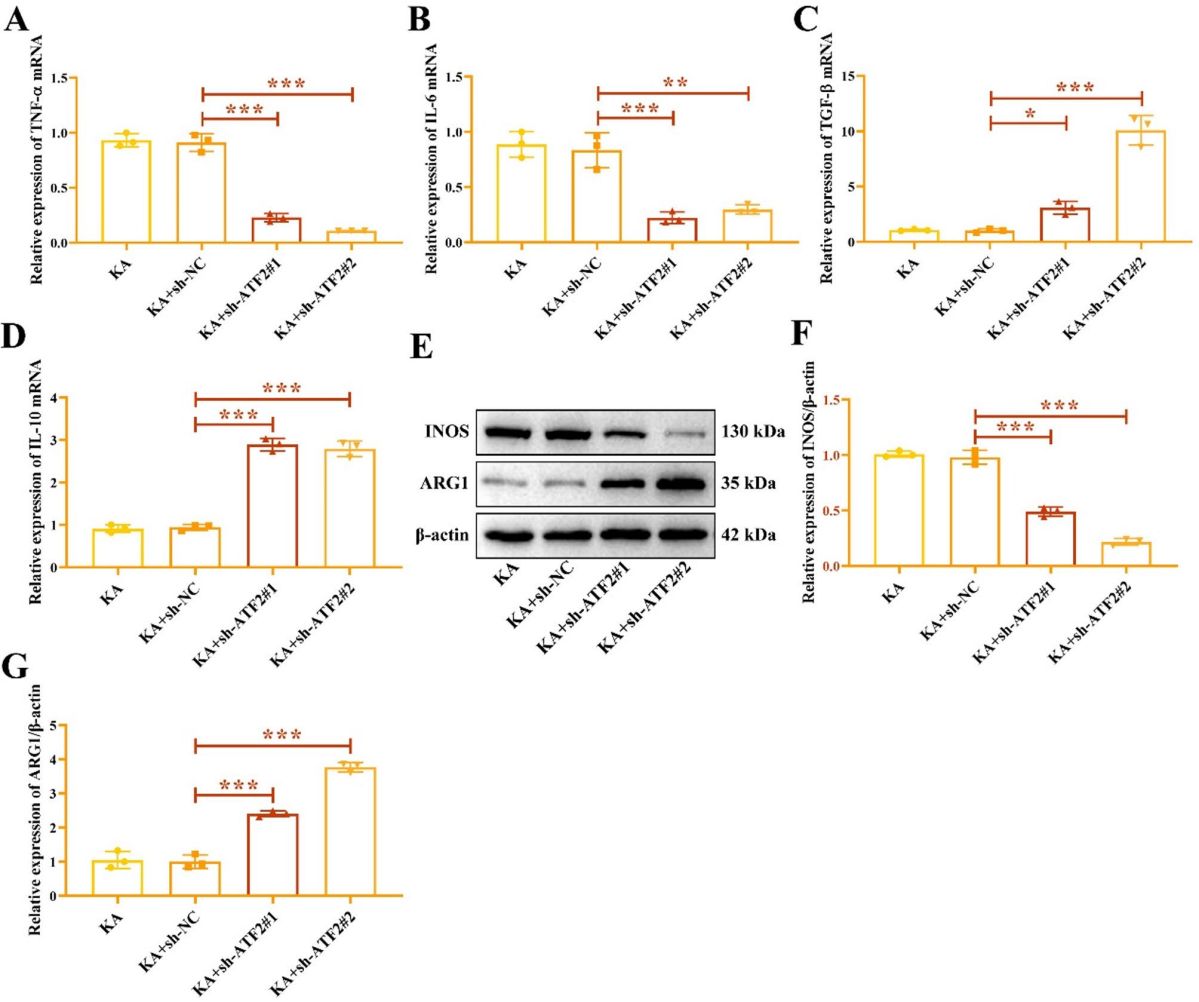
ATF2 inhibition represses the M1 polarization of HMC3 cells. The mRNA expression of TNF-α (**I**), IL-6 (**B**), TGF-β (**C**), and IL-10 (**D**) in HMC3 cells transfected with sh-ATF2. (**E**) The gel images of INOS and ARG1. (**F**) Relative expression of INOS protein. (**G**) Relative expression of ARG1 protein. ^*^P < 0.05, ^**^P < 0.01, ^***^P < 0.001.

RT-qPCR showed that the expression of pro-inflammatory cytokines TNF-α (Fig. 5A) and IL-6 (Fig. 5B) mRNA in ATF2 inhibition group was lower than that in sh-NC group, knocking down ATF2 promoted anti-inflammatory cytokines TGF-β (Fig. 5C) and IL-10 (Fig. 5D) mRNAs expression. Western blot results indicated that ATF2 inhibition decreased INOS protein (Fig. 5E-F), and promoted ARG1 protein (Fig. 5E and G). The original gels were shown in the Supplementary Fig. 5. Taken together, knockdown of ATF2 accelerates the M2 polarization of KA-induced HMC3 cells, but retards the M1 polarization.

### ATF2 regulates TSC1 transcription

**Fig. 6:**
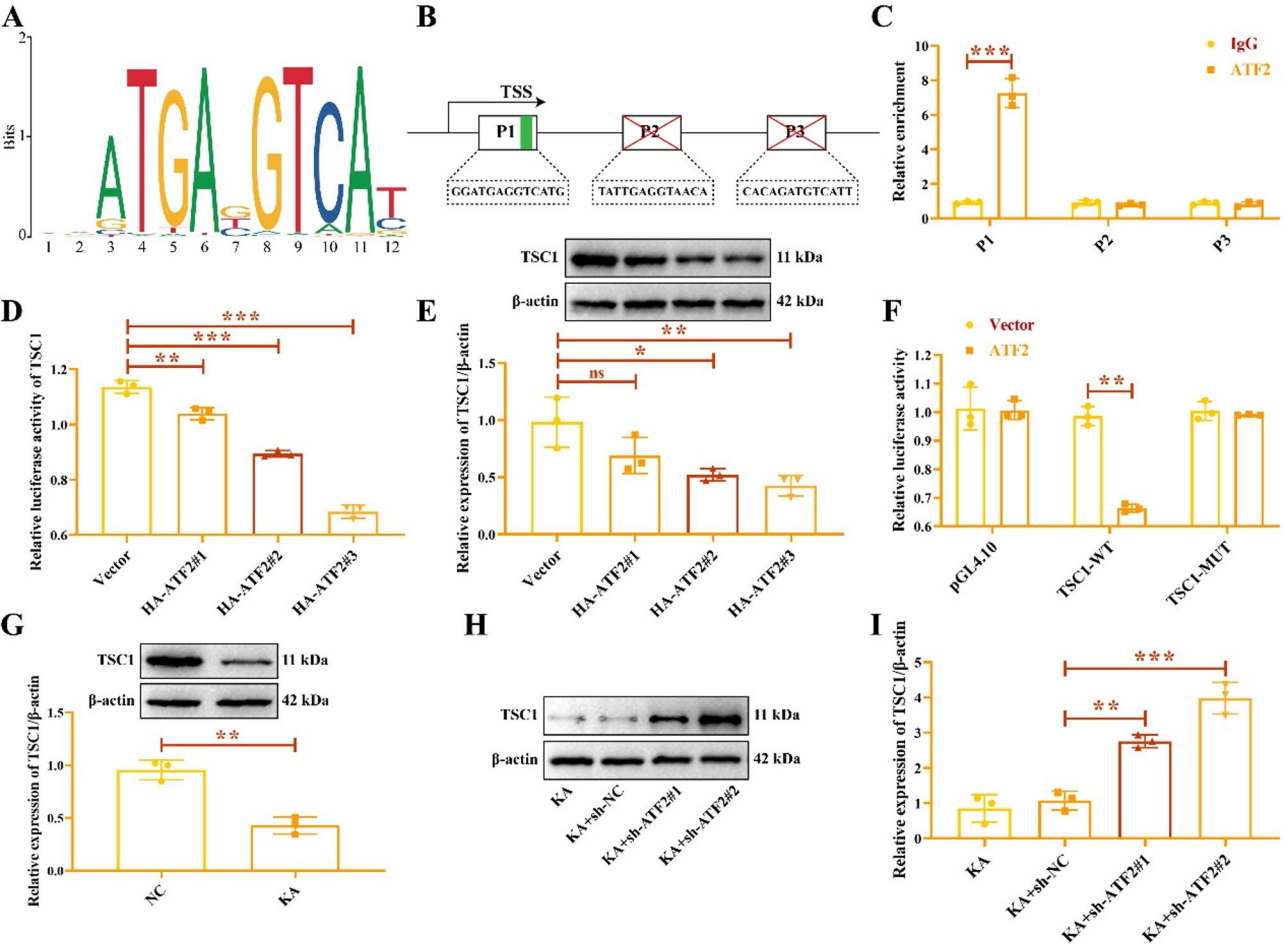
ATF2 regulates TSC1 transcription. (**A**) The binding sites of ATF2 obtained from JASPAR database. (**B**) The promoter regions (P1: promoter region 1, P2: promoter region 2, P3: promoter region 3) of TSC1. (**C**) The relative enrichment of ATF2 in TSC1 promoter regions. Dual luciferase reporter (**D**) and western blot (**E**) assay revealed that ATF2 inhibited the transcription of TSC1. (**F**) The luciferase activity of wild-type (WT) and mutant (MUT) TSC1 in HMC3 cells. (**G**) Relative expression of TSC1 in KA-induced HMC3 cells. (**H**-**I**) Transfection with sh-ATF2 enhanced TSC1 protein. ^*^P < 0.05, ^**^P < 0.01, ^***^P < 0.001.

Through predicting of hTFtarget database, we found that ATF2 may be used as a transcription factor to regulate TSC1. We obtained the binding site of ATF2 through JASPAR database (Fig. 6A), and obtained the TSC1 promoter sequences that bound to ATF2 (Fig. 6B). We designed three pairs of primer sequences in TSC1 promoter region. CHIP-PCR detection showed that ATF2 was significantly enriched in TSC1 promoter P1 (Fig. 6C). Subsequently, we transfected the empty vector and pcDNA3.1-HA-ATF2 into HMC3 cells, and found that ATF2 inhibited the luciferase activity (Fig. 6D) and protein expression of TSC1 in a dose-dependent manner (Fig. 6E). The original gels were shown in the Supplementary Fig. 6. In addition, overexpression of ATF2 repressed the luciferase activity of wild-type TSC1. However, ATF2 overexpression had no significant effect on luciferase activity of mutant TSC1 (Fig. 6F). Western blot was performed to detect TSC1 protein, and found that TSC1 protein was downregulated in KA-induced HMC3 cells than that in NC group (Fig. 6G). Knocking down ATF2 promoted TSC1 protein (Fig. 6H-I). Therefore, these results suggest that ATF2 inhibits the transcription of TSC1.

### Overexpression of TSCI represses M1 polarization of KA-induced HMC3 cells

**Fig. 7:**
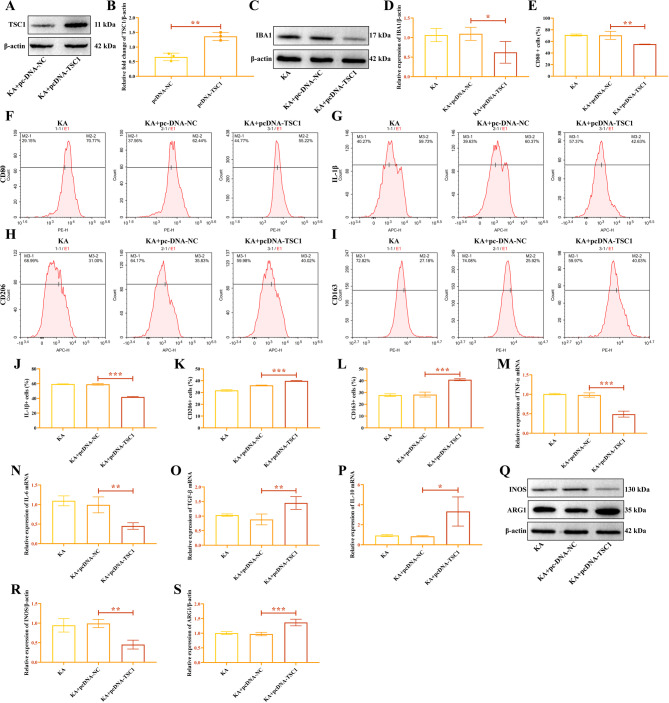
Overexpressed TSCI represses M1 polarization of KA-induced HMC3 cells. (**A**-**B**) TSC1 protein level in KA-stimulated HMC3 cells transfected with pcDNA-TSC1. (**C**-**D**) The relative expression of IBA1 protein. Flow cytometry detected the cell ratios of CD80 (**E**-**F**), IL-1β (**G** and **J**), CD206 (**H** and **K**), and CD163 (**I** and **L**). The relative expression of TNF-α (**M**), IL-6 (**N**), TGF-β (**O**), and IL-10 (**P**) mRNA. (**Q**) The gel images of INOS and ARG1. (**R**) Relative expression of INOS protein. (**S**) Relative expression of ARG1 protein. ^*^P < 0.05, ^**^P < 0.01, ^***^P < 0.001.

To investigate the role of TSCI in KA-induced HMC3 cells, we transfected the cells with pcDNA-NC and pcDNA-TSC1. The expression of TSC1 was elevated in HMC3 cells transfected with TSC1 (Fig. 7A-B). Compared to the overexpressed NC group, the expression of IBA1 protein was reduced in the TSC1 overexpression group (Fig. 7C-D), with the original IBA1 gel shown in Supplementary Fig. 7. Flow cytometry results indicated that overexpression of TSC1 downregulated the proportion of M1 macrophage markers CD80 (Fig. 7E-F) and IL-1β (Fig. 7G and J) positive cells, while promoting the proportion of M2 macrophage markers CD206 (Fig. 7H and K) and CD163 (Fig. 7I and L) positive cells.

RT-qPCR detection revealed that TNF-α and IL-6 mRNA expression were decreased in the TSC1 overexpression group (Fig. 7M-N), while TGF-β and IL-10 expression were increased (Fig. 7O-P). Compared to the overexpressed NC group, overexpression of TSC1 inhibited the expression of INOS (Fig. 7Q and R) and upregulated ARG1 protein expression (Fig. 7Q and S) in KA-induced HMC3 cells. These results suggest that overexpression of TSC1 can suppress macrophage M1 polarization and promote M2 polarization in KA-induced HMC3 cells, increasing the level of anti-inflammatory cytokines in the cells.

### ATF2 regulates microglia polarization in KA-induced mouse by medicating TSC1 transcription

Previous studies have found that ATF2 can regulate KA-induced microglia polarization through TSC1. Here, we established EP mouse models to investigate whether ATF2 regulates microglia polarization in EP mouse through TSC1 in vivo.

**Fig. 8:**
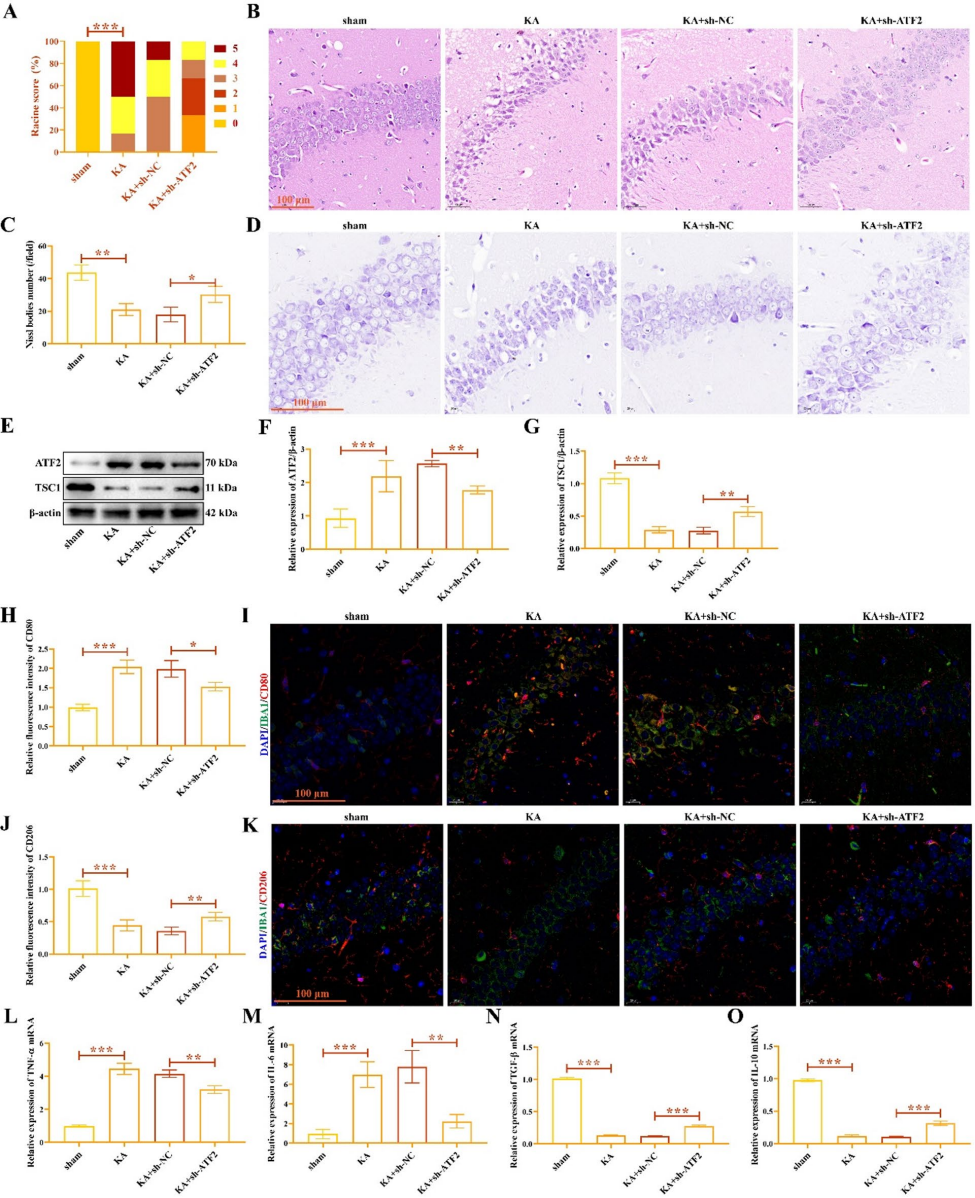
ATF2 regulates microglia polarization in KA-induced mouse by medicating TSC1 transcription. (**A**) Racine scale scores were used to evaluate the seizure severity of mice. (**B**) HE staining of mouse hippocampal tissues. (**C**-**D**) Nissl staining of mouse hippocampal tissues. (**E**) The gel images of ATF2 and TSC1. The relative expression of ATF2 (**F**) and TSC1 (**G**). (**H**-**I**) The fluorescence staining of IBA1 and CD80. (**J**-**K**) The fluorescence staining of IBA1 and CD206. The relative expression of TNF-α (**L**), IL-6 (**M**), TGF-β (**N**), and IL-10 (**O**). ^*^P < 0.05, ^**^P < 0.01, ^***^P < 0.001.

The scores of Racine scale in EP group were all greater than 2, and the severity of epileptic seizures in ATF2 knockdown group decreased (Fig. 8A). The results of HE staining in mouse hippocampal tissue were shown in Fig. 8B. In sham group, the morphology and structure of hippocampus were normal, the cells were arranged neatly, and the size of nucleus and nucleolus was normal. In EP model group, the cells in hippocampus were loosely arranged and disordered, and the nuclei were reduced or lost. In ATF2 knock-down group, the damage of hippocampal neurons was relieved, and cell vacuolation was reduced. The number of Nissl staining positive cells in EP group was less than that in sham group (Fig. 8C-D), and ATF2 knocking down significantly increased the number of Nissl staining positive cells.

Compared with sham mouse, ATF2 protein was overexpressed (Fig. 8E-F) and TSC1 protein was decreased (Fig. 8E and G) in EP mouse, ATF2 inhibition repressed ATF2 protein and increased TSC1 protein in hippocampal tissues of mouse. The original gels were shown in the Supplementary Fig. 8. The fluorescence intensity of CD80 was enhanced in EP mouse than that in sham mouse (Fig. 8H-I), and ATF2 inhibition weakened CD80 fluorescence intensity. The fluorescence intensity of CD206 was inhibited in KA-induced mouse (Fig. 8J-K), whereas knocking down ATF2 enhanced the fluorescence intensity of CD206. The mRNA levels of pro-inflammatory cytokines TNF-α (Fig. 8L) and IL-6 (Fig. 8M) were elevated by stimulating of KA, anti-inflammatory cytokines TGF-β (Fig. 8N) and IL-10 (Fig. 8O) mRNA were inhibited. However, the mRNA level of TNF-α, IL-6, TGF-β, and IL-10 caused by KA could be were reversed by ATF2 inhibition. These data indicate ATF2 inhibition dampened the M1 polarization of microglia through promoting TSC1 expression, and improved the nerve injury of EP mouse.

## Discussion

EP is a brain dysfunction caused by abnormal excitation of neurons, which is characterized by spontaneity and recurrence. Neuroinflammation is an important mechanism for the occurrence and development of EP, and inflammatory reaction can promote the apoptosis of hippocampal neurons^22^. Microglia is the first line of defense, and its activation degree is closely related to the accumulation of inflammatory factors^20^. In the present study, we found that KA induced M1 polarization of HMC3 cells by activating KA receptors, and inhibiting KA receptors could attenuate KA-induced M1 polarization of HMC3 cells. ATF2 was overexpressed in KA-induced HMC3 cells and mouse. Subsequent investigation revealed that knocking down ATF2 repressed M1 polarization and the expression of pro-inflammatory cytokines, induced the M2 polarization and the expression of anti-inflammatory cytokines through promoting the transcription of TSC1. These fundings suggest that ATF2 inhibition may be a potential target for EP therapy.

Microglia play an important role in the development of EP by supporting and regulating neuronal activity^23^. Beach et al.^24^ found in autopsy that the number of HLA-DR-immunoreactive microglia in hippocampal CA1 and CA3 areas of epileptic patients was significantly higher than that in the control group. The duration and frequency of EP are obviously related to microglia activation^25^. The ablation of microglia represses the activation of astrocytes and its complement C3a receptor, and reduces the glial hyperplasia and neuronal damage in epileptic mouse^26^. Knocking out TRPM2 in microglia weakens the activation of glia, the production of inflammatory cytokines, the paroxysmal discharge of hippocampus, and alleviates the neuroinflammation induced by EP^27^. The absence of TAK1 significantly reduces the number and reactivity of microglia in hippocampus of epileptic mouse and the chronic epileptic activity^28^. GSDMD knockdown in microglia weakens the phagocytic activity of microglia and increases the susceptibility to EP and neuronal apoptosis^29^. Our results indicated that inhibition of ATF2 suppressed the M1 polarization of KA-induced HMC3 and reduced neuroinflammation in EP mouse by promoting the transcription of TSC1.

ATF2 is an important component of activator protein-1 transcription complex, which can be used as a transcription factor to regulate cell growth and development, oxidative stress, cell cycle and apoptosis. The abnormal expression and function of ATF2 is closely related to the occurrence of tumors and other diseases, and it has the activity of promoting or inhibiting diseases in different cells and tissues^30,31^. Previous studies indicates that ATF2 overexpression promotes the sensitivity of macrophages to M1 polarization, and enhances chemokine-rich signal transduction, metabolism and antigen presentation^32^. ATF2 expression and its phosphorylation level are up-regulated in synovial tissue of rheumatoid arthritis, knockdown of ATF2 dampened the migration and invasion of fibroblast-like synovial cells in osteoarthritis, reduces the production of inflammatory cytokines^33^. ATF2 inhibition represses the glycolysis level and pyroptosis of microglia induced by lipopolysaccharide^34^. Inhibition of ATF2 signaling pathway weakens neuroinflammation mediated by microglia, inhibits apoptosis of hippocampal neurons, and alleviates perioperative neurocognitive disorder of mouse^12^. We found that ATF2 expression was upregulated in KA-induced HMC3 cells and mouse; knocking down ATF2 repressed the M1 polarization of HMC3 cells, decreased the expression of TNF-α and IL-6, accelerated the M2 polarization of microglia, and improved the nerve injury of EP mouse.

TSC1 is located on chromosome 9q34 and consists of 21 exons. TSC2 is located on chromosome 16p13 and consists of 41 exons. TSC1 and TSC2 can inhibit the mechanistic target of rapamycin complex 1 (mTORC1) by inhibiting Rheb activity. In the resting state, TSC1 maintains the RAS family GTPase Rheb in an inactive state, the GDP-bound form. When stimulated by external signals, TSC1 exerts a biological regulatory effect by promoting the direct upstream activation factor Rheb GTP of mTORC1 to convert into Rheb GDP. Tuberous sclerosis complex is an autosomal dominant monogenic disease, which is caused by the abnormal structure and function of TSC1/TSC2 complex caused by TSC1 or TSC2 mutation. According to the statistics, about 80% patients with tuberous sclerosis complex are accompanied by EP^35^. Abs et al.^36^. found that mouse developed epileptic symptoms a few days after the deletion of the double allele TSC1, and the activity of TORC1 was reduced and the epileptic symptoms were eliminated after rapamycin treatment. In the TSC1 conditional knockout mouse model, the inactivation of TSC1 in the late embryonic radial glial leads to social and cognitive dysfunction and spontaneous seizures in mouse^37^. Our results indicated that ATF2 regulated the transcription of TSC1; overexpression of TSC1 can alleviate the promotion of overexpression of ATF2 on M1 polarization of KA-induced HMC3 cells, induce M2 polarization of HMC3 cells. Moreover, Shi et al.^38^ found that the combined deletion of TSC1 and Trem2 led to the decrease of Aβ clearance and the increase of Aβ plaque burden, which induced cognitive dysfunction in mouse with Alzheimer’s disease. In TSC1 knockout mouse, the activation of mTORC1 promotes the activation of microglia and accelerates the aging and degeneration of retina^39^.

In conclusion, our results indicate that ATF2-TSC1 medicates the neuroinflammation of EP. Knockdown of ATF2 promotes the transcription of TSC1, represses neuroinflammation mediated by microglia to improve EP. Whether TSC1 mediates the development of EP by regulating mTOR signaling pathway is still unclear, which needs to be explored in the follow-up study.

## Electronic supplementary material

Below is the link to the electronic supplementary material.

Supplementary Material 1

## Data Availability

Data is provided within the manuscript or supplementary information files.
