# Supplementary material for: Effects of ATF2/TSC1 on epilepsy by modulating the microphages polarization of microglia
Source: Sci Rep. 2025 Jul 2;15:22958. doi: 10.1038/s41598-025-04914-4 (PMC12214619; doi:10.1038/s41598-025-04914-4)
Supplement: Supplementary file 1 — Supplementary Material 1 [file 41598_2025_4914_MOESM1_ESM.docx]

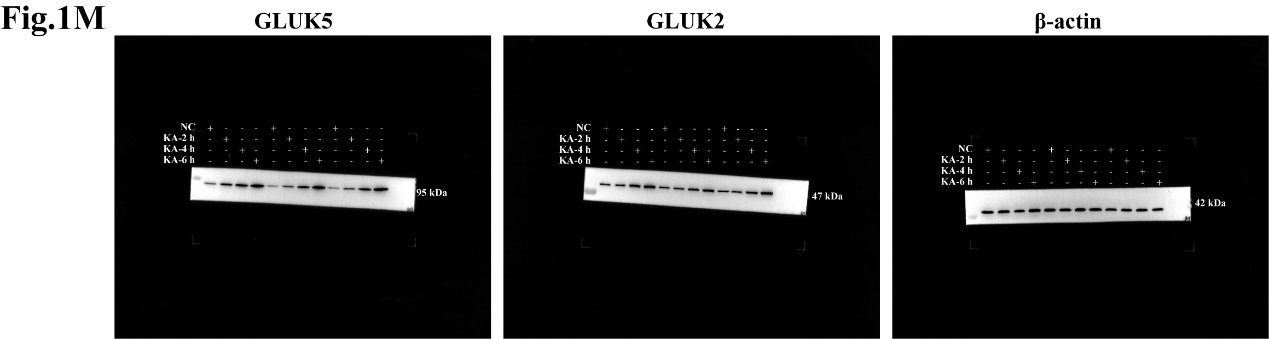


**Supplementary Figure 1**. The original western blot images corresponding to Fig.1M.


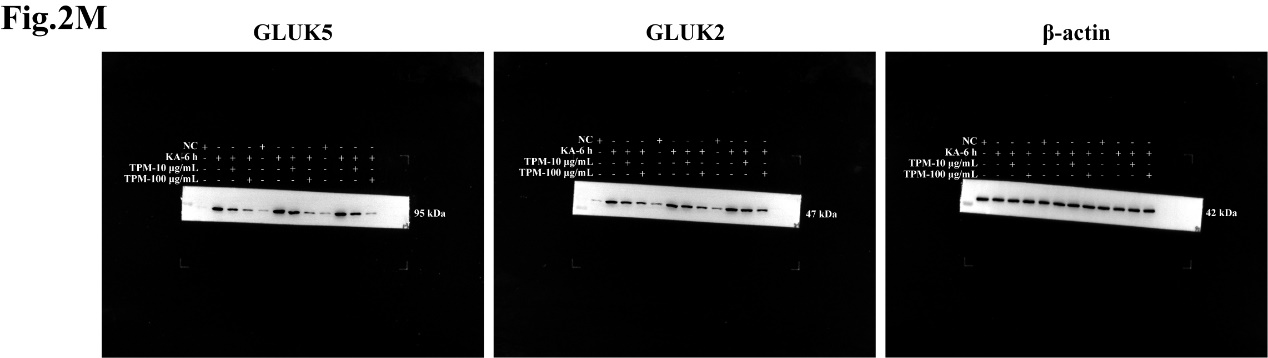


**Supplementary Figure 2**. The original western blot images corresponding to Fig.2M.


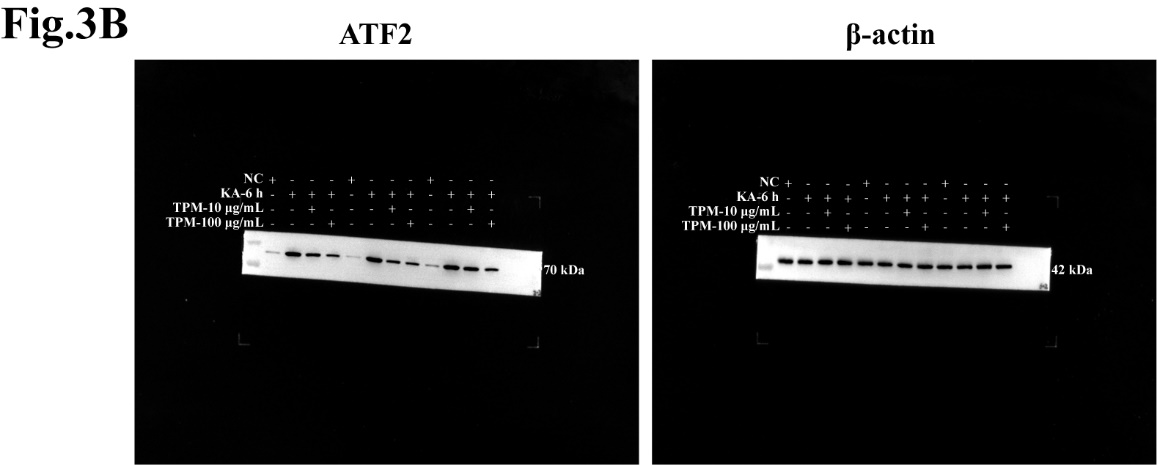


**Supplementary Figure 3**. The original western blot images corresponding to Fig.3B.

**
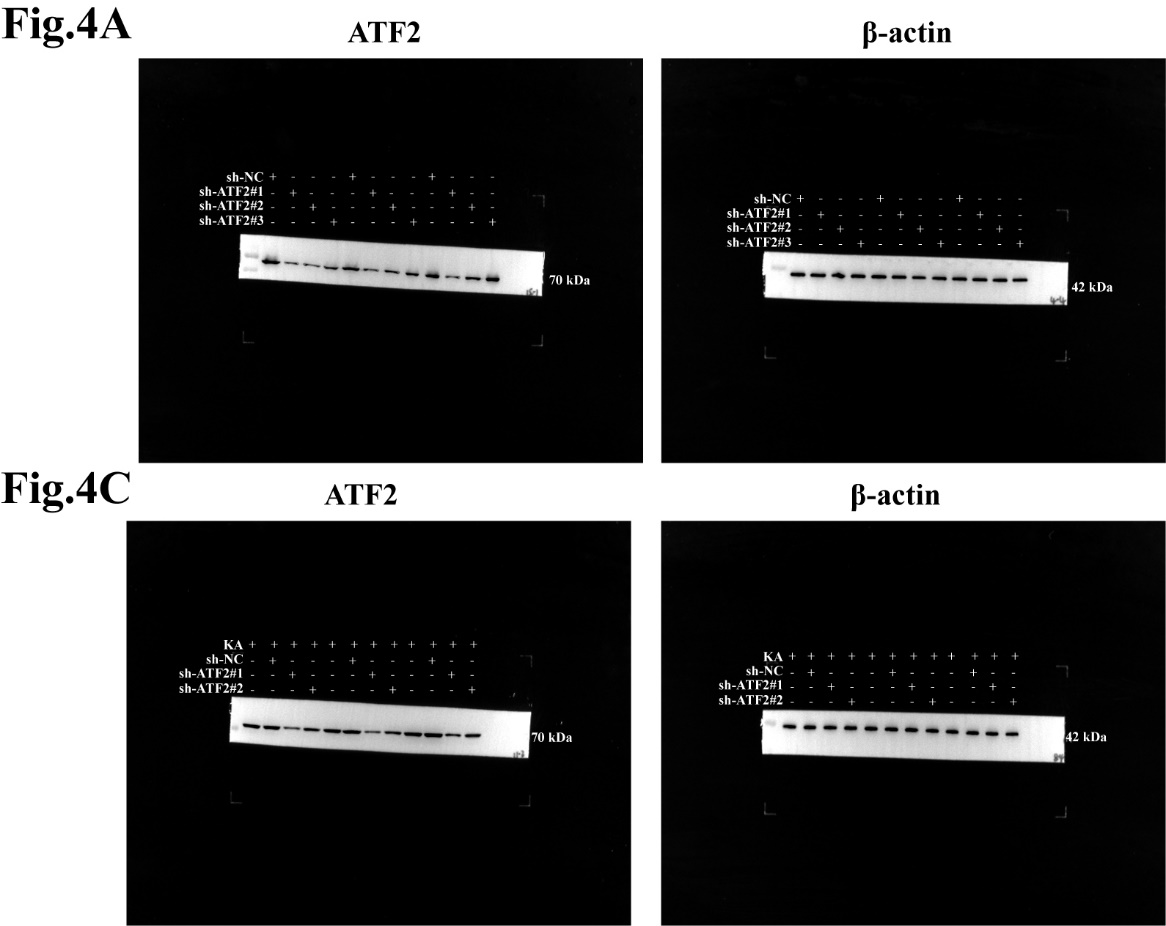
**

**Supplementary Figure 4**. The original western blot images corresponding to Fig.4A and 4C.


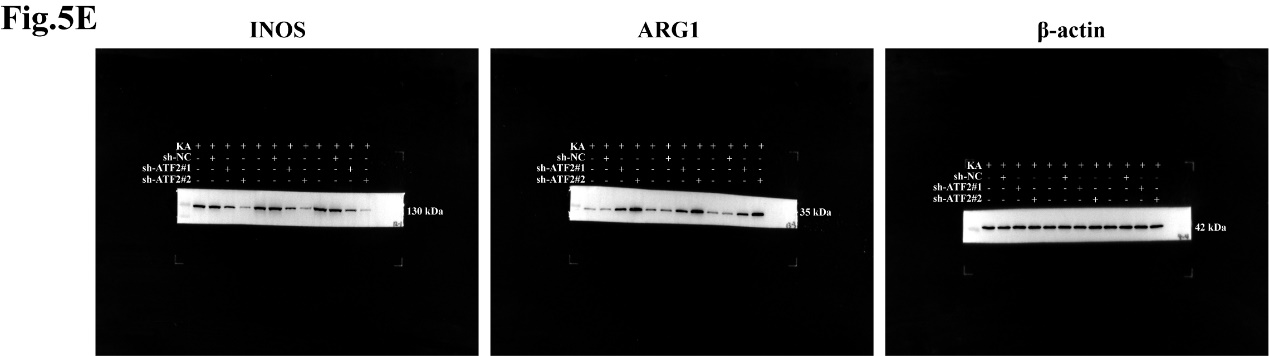


**Supplementary Figure 5.** The original western blot images corresponding to Fig.5E.


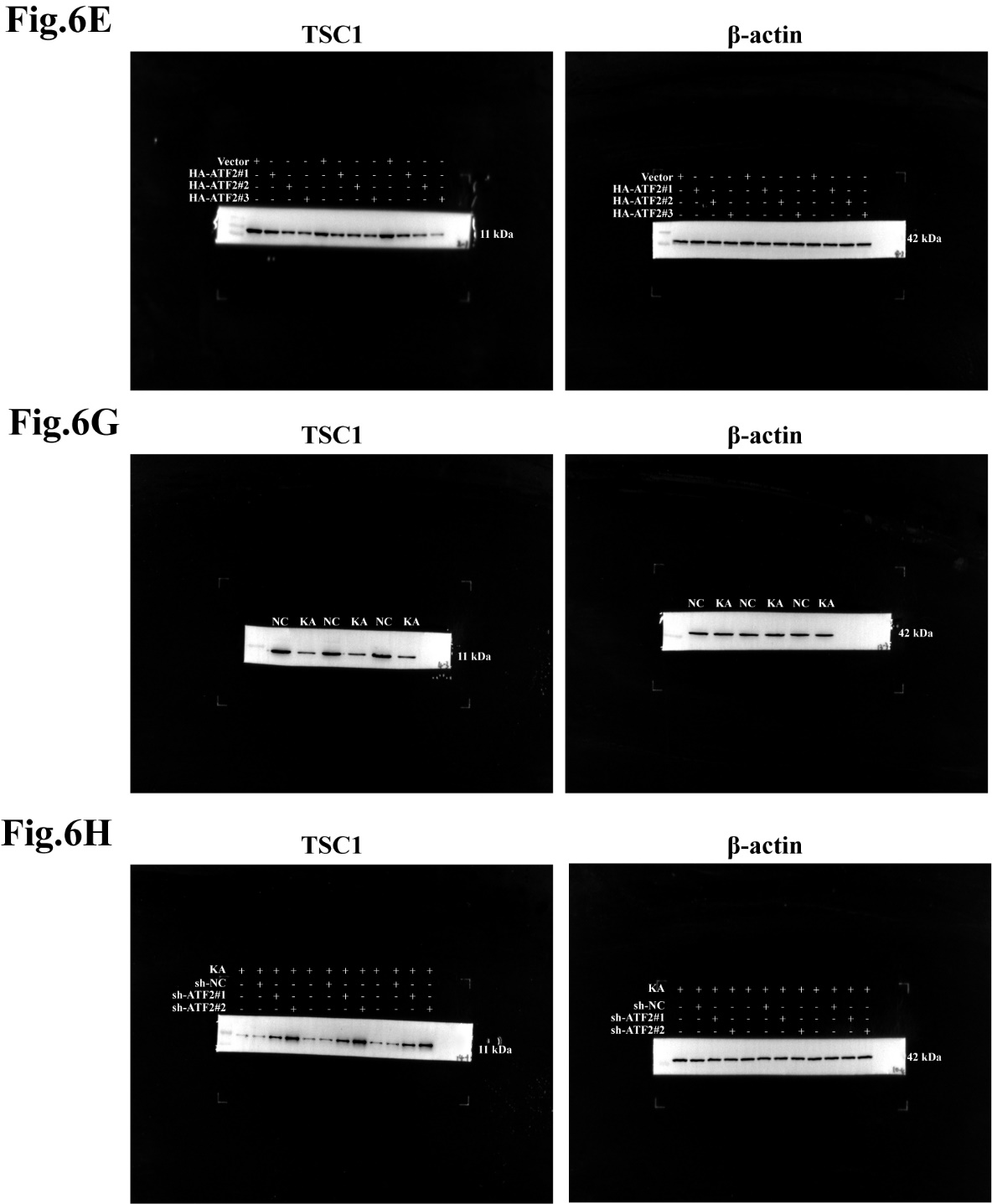


**Supplementary Figure 6**. The original western blot images corresponding to Fig.6E, 6G, and 6H.


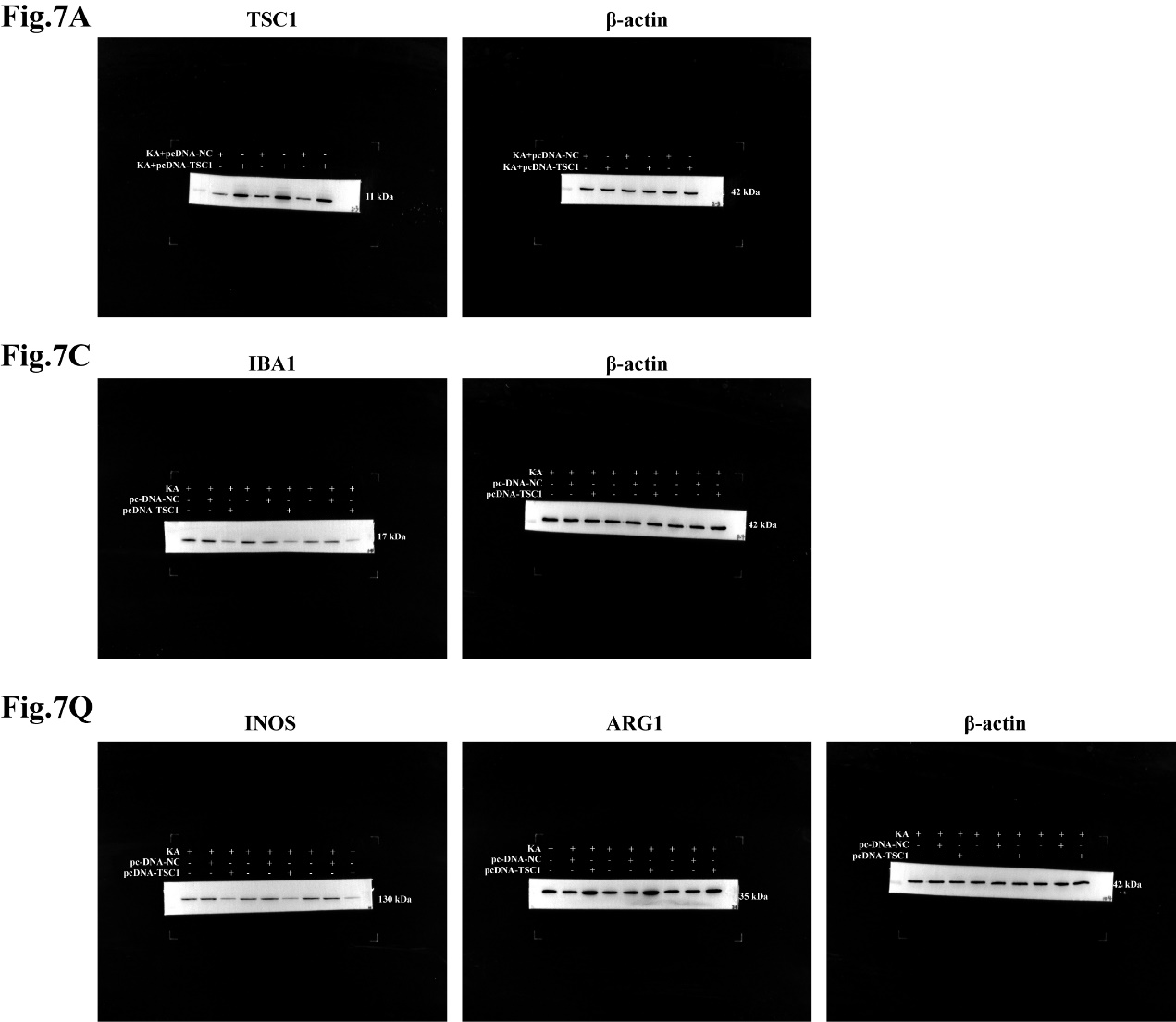


**Supplementary Figure 7**. The original western blot images corresponding to Fig.7A, 7C, and 7Q.


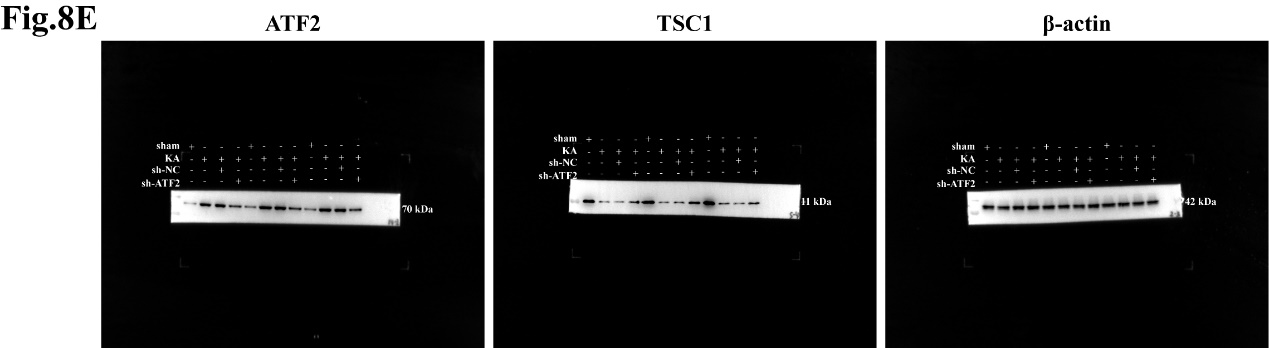


**Supplementary Figure 8**. The original western blot images corresponding to Fig.8E.
